# Acute and Subchronic Oral Toxicity Evaluation of Herbal Formulation: *Piper crocatum* Ruiz and Pav., *Typhonium flagelliforme* (Lodd.) Blume, and *Phyllanthus niruri* L. in Sprague–Dawley Rats

**DOI:** 10.1155/2023/7511397

**Published:** 2023-01-03

**Authors:** Retno Murwanti, A. Nurrochmad, Andayana P. Gani, Ediati Sasmito, Angela E. Edwina, Mayang K. Chandra, F. H. Suryawan, A. R. Wardana, Natalia, Jelita L. S. R. Budiningsih

**Affiliations:** ^1^Department of Pharmacology and Clinical Pharmacy, Faculty of Pharmacy, Universitas Gadjah Mada, Yogyakarta, Indonesia; ^2^Medicinal Plants and Natural Products Research Center, Faculty of Pharmacy, Universitas Gadjah Mada, Yogyakarta, Indonesia; ^3^Department of Pharmaceutical Chemistry, Faculty of Pharmacy, Universitas Gadjah Mada, Yogyakarta, Indonesia; ^4^Department of Pharmaceutical Biology, Faculty of Pharmacy, Universitas Gadjah Mada, Yogyakarta, Indonesia

## Abstract

**Background:**

The product combination of *Piper crocatum* Ruiz. and Pav., *Phyllanthus niruri* Linn., and *Typhonium flagelliforme* (Lodd.) BL ethanolic extract (SKM) exerts immunomodulatory activity. However, the toxicity profile of the combination has never been investigated.

**Objective:**

This study aimed to establish the acute toxicity profile of the SKM product on Sprague–Dawley (SD) rats and its subchronic toxicity profile on female SD rats.

**Method:**

The acute and subchronic toxicity tests were conducted in accordance with OECD 423 and OECD 408, respectively.

**Result:**

The SKM product was safe up to 5000 mg/kg b.w. in male and female SD rats. In repeated doses of SKM for 90 days, the administration of 22.5, 45, and 90 mg/kg b.w. per day of the SKM product to female SD rats did not affect clinical signs, body weight, food and water consumption, hematological parameters, clinical chemical parameters, urinalysis, relative organ weights, and gross pathological and histopathological features compared with the control group.

**Conclusion:**

Analyses of these results suggest that the long-term oral administration of the SKM product for 90 days does not cause subchronic toxicity.

## 1. Introduction

Single extracts of *Piper crocatum* Ruiz and Pav. leaf (*P. crocatum*) [1], *Typhonium flagelliforme* (Lodd.) Blume tuber (*T. flagelliforme*) [2], and *Phyllanthus niruri* L. herbs (*P. niruri*) [3] exert immunomodulatory activity. Ariefqiani found that combining *P. niruri*, *T. flagelliforme*, and *P. crocatum* can improve lymphocyte proliferation [4]. Thus, this combination can be considered as a cellular immunostimulator.

An acute toxicity study showed that the lethal dose 50 (LD50) of *P. niruri* ethanolic extract in mice is 18,000 mg/kg b.w. [5]. In addition, the lethal dose of *T. flagelliforme*, which causes 50% of death, is 48.081 g/kg b.w. [6]. Halim performed an acute toxicity study using Balb/c mice and found that the LD50 value of *P. crocatum* extract is 1,588,781 mg/kg b.w. [7].

Several studies examined the toxicity profile of each plant. Hartini et al. showed that neolignan Pc-2 isolated from *P. crocatum* leaves demonstrates hydropic degeneration and histopathological features of necrosis [8]. The Brine Shrimp Lethality Test results demonstrated that *T. flagelliforme* tuber hexane extract is slightly toxic, with an LD50 value of 762,086 ppm [6].

A toxicity study reported that *P. niruri* extract has no toxic effect on aminotransferase (AST) and alanine aminotransferase (ALT) levels in diabetes mellitus rats at 50 and 100 mg/kg b.w. [9]. A subchronic toxicity test showed that the ethanolic extract combination of *Piper crocatum* leaves, *Typhonium flagelliforme* Lodd. Blume. tuber, and *Phyllanthus niruri* Linn. herbs is nontoxic on liver function [10].

However, the toxicity profile of combined extracts remains unknown. Therefore, the potential acute and subchronic toxicity profiles of combined extracts in Sprague–Dawley (SD) rats should be investigated. This study analyzed the acute and subchronic safety profiles of the combination product of *P. crocatum*, *P. niruri*, and *T. flagelliforme* ethanolic extract (SKM product).

## 2. Materials and Methods

### 2.1. Materials

SKM product contains ethanolic extract of *P. niruri* leaves (75 mg), ethanolic extract of *T. flagelliforme* tubers (100 mg), ethanolic extract of *P. crocatum* herbs (75 mg), Amprotab® (amylum manihot, 100 mg), and Compercell® (microcrystalline cellulose, 100 mg).

### 2.2. Experimental Animals

Male and female SD rats aged 6–8 weeks old with an initial body weight of 100–150 g were used for the acute toxicity study, and only female rats were used for the subchronic study. Female rats were selected based on the OECD guideline test No. 423 and No. 408 precisely due to the better sensitivity of female rats than males. All the animals for the acute toxicity study were obtained from the Laboratory of Pharmacology and Toxicology, Faculty of Pharmacy, Universitas Gadjah Mada, Indonesia. The subchronic study used female rats supplied by the Animal Experimental Unit, Animal Research, and Development Center, Universitas Gadjah Mada. SD rats were randomly divided, marked, and acclimated to laboratory conditions. The rats were acclimatized for 5 days to avoid stress before administration. The temperature was set to approximately 25 C–30 C. Humidity was about 40%–70%, and the light condition was 12 h bright and 12 h dark. Temperature and humidity were controlled using an air conditioner, a room thermometer, and a room hygrometer. This study was approved by the Ethical Committee for Preclinical Study, Central University Laboratory, Universitas Gadjah Mada (Acute Toxicity Study Registration No. 316/KEC-LPPT/IX/2015; Subchronic Toxicity Study Registration No. 310/KEC- LPPT/VIII/2015).

### 2.3. Acute Toxicity Study

The study was conducted in accordance with OECD Guidelines test number 423 [11]. SKM product was suspended in 0.5% sodium CMC solvent and administered to the rats by single gavage doses after 24 h fasting. The animals were divided into three groups of six rats each: control, 2000 mg/kg b.w. dose, and 5000 mg/kg b.w. dose. The rats from different groups were placed in different cages. Cages were plastic boxes with chaff as the base.

The rats in the control group received sodium CMC solvent only to evaluate the effect of the vehicle. The initial dose in this study was 2000 mg/kg b.w., considering the acute toxicities of every single extract. All deviations in general behaviors were observed for 4 h after dosing, and then the monitoring was conducted daily for 14 consecutive days. Body weights were measured before and after administration daily. Necropsy was performed on all animals at the end of the 14-day observation to find apparent changes in major organs and tissues.

### 2.4. Subchronic Toxicity

The study was performed following OECD Guidelines test number 408 [12]. Forty rats were randomly separated into four groups of 10 each: control (received CMC-Na 0.5% as a vehicle), 22.5 mg/kg b.w., 45 mg/kg b.w., and 90 mg/kg b.w. Additional 10 rats for the recovery period were divided into the satellite control group and the satellite 90 mg/kg b.w. group. The test material was suspended with CMC-Na 0.5% and orally administrated for 90 consecutive days. The test material was mixed with a vehicle every 5 days. All animals were observed for clinical signs and food and water consumption once daily. Individual body weights were recorded every 3 days. Rats in control and dose groups were sacrificed at day 90. Satellite groups were continuously observed without treatment in the next 28 days for the recovery period and then sacrificed.

#### 2.4.1. Hematology and Clinical Chemistry

Blood samples for hematology and clinical chemistry assessments were drawn from the infraorbital venous and placed in a vacutainer tube containing EDTA. The hematological parameters, including red blood cell (RBC) count, white blood cell (WBC) count, hemoglobin (Hb), hematocrit (HCT), mean corpuscular volume (MCV), mean corpuscular hemoglobin (MCH), mean corpuscular hemoglobin concentration (MCHC), and blood platelet count (PLT), were evaluated on an automatic hematology analyzer Sysmex KX-21. Clinical chemistry parameters, including serum AST, serum ALT, total protein (TP), total cholesterol (TC), glucose (GLU), urea (UREA), and creatinine (CRE), were analyzed with Merck Photometer type Microlab 300.

#### 2.4.2. Urinalysis

Urine was collected for 24 h using metabolic cages. The animals fasted overnight, but the water was provided ad libitum during the collection period. Volume, pH, and color were observed.

#### 2.4.3. Organ Weight and Histopathological Evaluation

The relative organ weights of the heart, spleen, liver, lungs, kidneys, stomach, and intestine were calculated by dividing organ weight by body weight. The organs were washed in an isotonic solution, fixed in 10% formalin, and embedded in paraffin to ease the slicing. The samples were stained with hematoxylin-eosin dye and then examined microscopically.

### 2.5. Statistical Analysis

Quantitative data were presented as mean value ± standard deviation. All results of the treatment groups were compared with those of the control group. Data were analyzed using SPSS 16.0 for Windows (SPSS Inc., Chicago, IL, USA). Statistical methods were used to analyze the body weight, relative organ weight data, food and water consumption, hematological parameters (RBC count, WBC count, Hb, HCT, MCV, MCH, MCHC, and PLT), and biochemical parameters (AST, ALT, TP, TC, GLU, UREA, and CRE). The one-way ANOVA was used to show differences among the groups, whereas a paired sample *T*-test was used to show significant differences between days 0 and 90. An independent sample *T*-test was used to show significant differences between days 90 and 118. Statistical significance was considered at *P* < 0.05.

## 3. Results

### 3.1. Acute Oral Toxicity

#### 3.1.1. Total of Death and Toxic Syndrome

The animals used in the main study were observed 14 consecutive days after dosing, and none showed any toxic signs or died. No male or female rats died during the 14 days of observation. The general appearance, grooming, posture, gait, behavior, and all other observational parameters were normal during the study.

#### 3.1.2. Body Weight

**Figure 1:**
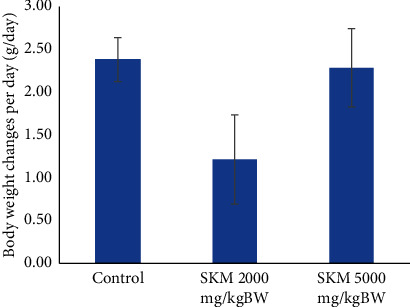
Average changes in body weight of SD male rats in 14 days.

**Figure 2:**
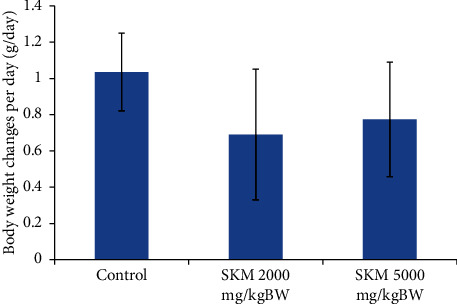
Average changes in body weight of SD female rats in 14 days.

Body weight data were presented as average changes in body weight. It is one of the parameters to evaluate the general health condition of animal experiments. It was obtained by reducing the body weight of test animals on the 14th day with body weight on the first day and then dividing by 14. The average of the body weight changes is shown in Figures 1 and 2. No significant difference in body weight was found between the treated and control groups.

#### 3.1.3. Organ Weight

Organ weight data were expressed as a relative weight (Tables 1 and 2). No significant difference in organ weight was found between the treated and control groups (*P* > 0.05).

These results of the animal experiments indicate that the SKM product does not cause any changes in the relative weights of organs. The average relative organ weights of male and female animals are shown in Tables 1 and 2.

#### 3.1.4. Histopathological Evaluation

**Figure 3:**
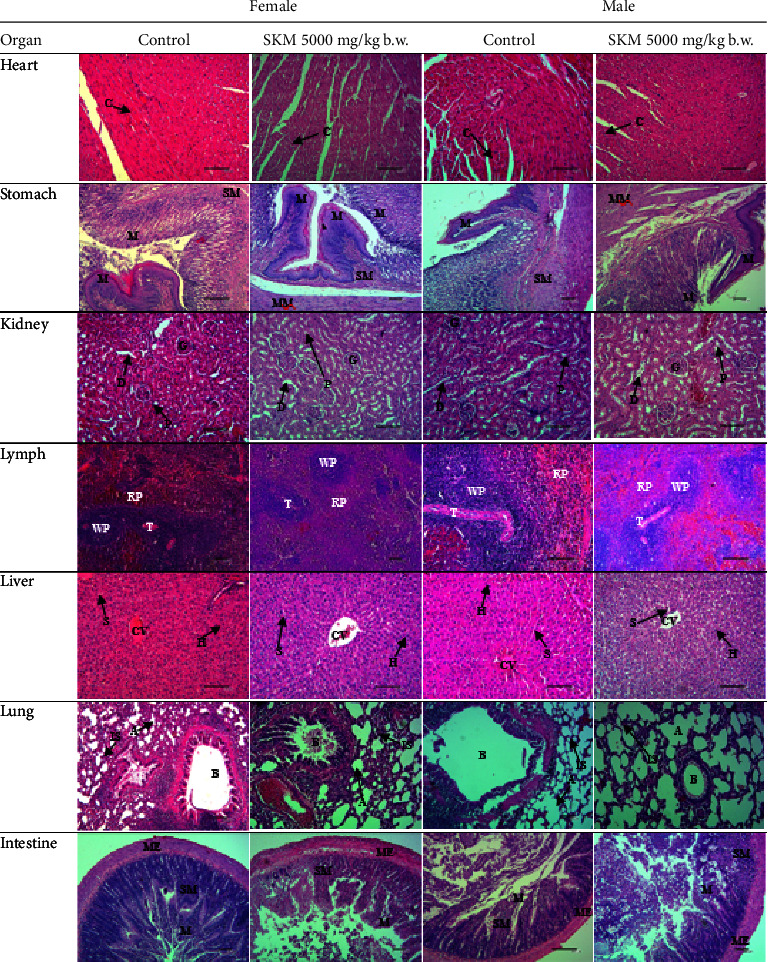
Hematoxylin and eosin-stained histological sections of the heart, kidney, lymph, liver, lung (200x magnification), stomach, and intestine (100x magnification) of the control rats and those exposed to 5000 mg/kg b.w. SKM acutely. No significant alteration was observed in all treatment groups. Cardiomyocyte bundle (C), mucosa (M), submucosa (SM), muscularis mucosa (MM), proximal tubules (P), distal tubules (D), glomerulus (G), white pulp (WP), red pulp (RP), trabeculae (T), sinusoid (S), central veins (CV), hepatocyte (H), alveolus (A), bronchiole (B), interalveolar septa (IS), and muscularis externa (ME).

Histological examination showed no morphological alteration for 14 days (Figure 3).

### 3.2. Subchronic Oral Toxicity

#### 3.2.1. Clinical Signs and Mortality

No toxicity symptom or death related to SKM administration was observed during the treatment and recovery periods at any dose.

#### 3.2.2. Body Weight

**Figure 4:**
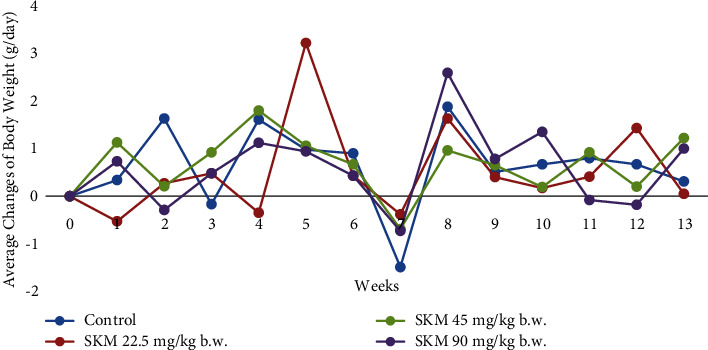
Average changes in body weight (g/day) of female rats exposed to SKM product (22.5; 45; 90 mg/kg b.w.) by oral route and control rats during the 90-day followed by a 28-day recovery period.

Fluctuated mean body weights were noted for all groups from week 2 (Figure 4). Reduction in mean body weights occurred at week 7 and rose again at week 8. No significant difference in the mean body weights during the experimental period was observed. Furthermore, the observed differences in body weight were not of toxicological relevance.

#### 3.2.3. Food and Water Consumption

**Figure 5:**
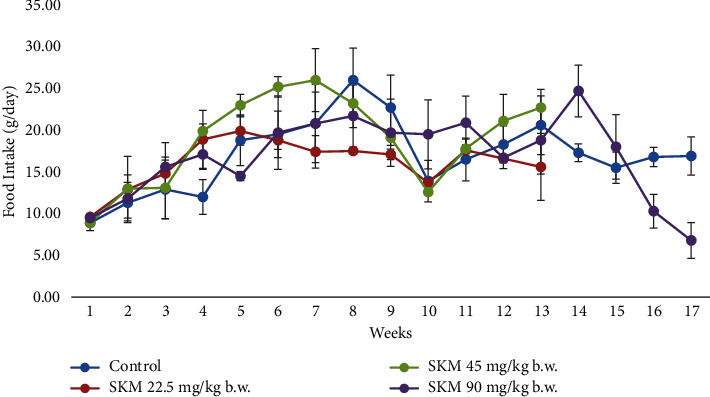
Food consumption (g/day) of SD female rats exposed to SKM product (22.5; 45; 90 mg/kg b.w.) by oral route and control rats during the 90-day followed by a 28-day recovery period.

**Figure 6:**
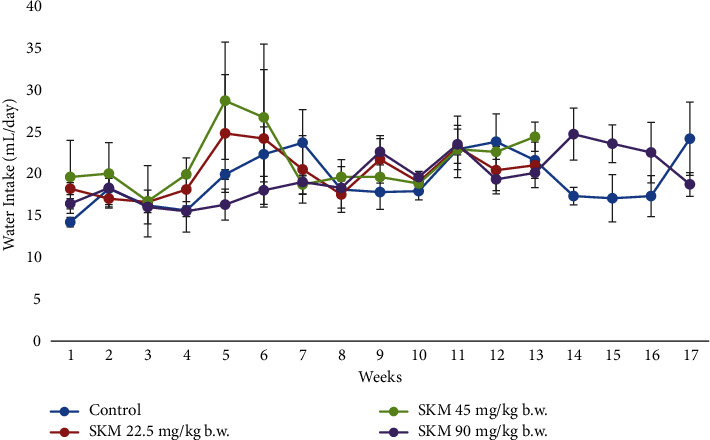
Water consumption (mL/day) of SD female rats exposed to SKM product (22.5; 45; 90 mg/kg b.w.) by oral route and control rats during the 90-day followed by a 28 day recovery period.

Overall, food and water consumption varied for all groups (Figures 5 and 6). However, the variation observed was similar and showed no attributable changes after the administration of the product.

#### 3.2.4. Hematology

The hematological changes are shown in Table 3. No significant changes were observed in comparison with the control group. By the end of the recovery period, no treatment-related differences were detected in these hematological parameters.

#### 3.2.5. Clinical Chemistry

Clinical chemistry or biochemical parameters of female SD rats exposed to SKM product (22.5; 45; 90 mg/kg b.w.) by oral route and control rats during the 90-day followed by a 28-day recovery period are shown in Table 4. There were no significant differences in ALT, AST, GLU, TC, urea, and CRE levels between the dose and control groups. Significant lower levels of TP occurred in the 90 mg/kg b.w. day 90 group.

#### 3.2.6. Urinalysis

Urinalysis did not indicate any treatment-related changes in the volume, pH, and colors. Several observed changes were considered incidental (shown in Table 5).

#### 3.2.7. Organ Weights

Relative organ weight changes are shown in Table 6. Statistical analysis showed no significant differences in relative liver, spleen, stomach, and heart weights compared with the control group. Significant differences in relative lung weight were found between the 45 mg/kg b.w. and control groups.

#### 3.2.8. Gross Pathology and Histopathology

In the gross pathological and histopathological examination, the appearance of the heart, lung, spleen, stomach, kidney, liver, and intestine was normal in all groups.

## 4. Discussion

For centuries, natural products, such as medicinal plants, have been the basis for the treatment of various ailments [15]. This research tries to reveal the effect of several natural products, specifically from Indonesia. It is an SKM product that contains the combination of *Piper crocatum* Ruiz. and Pav., *Phyllanthus niruri* Linn., and *Typhonium flagelliforme*. *Typhonium flagelliforme* is a medicinal plant native to Indonesia and can be used as an immunomodulator, antioxidant, and hepatoprotective agent. In addition, *Piper crocatum* Ruiz. and Pav. and *Phyllanthus niruri* Linn are the natural products that are originally from Asia. These plants have an activity to modulate immune responses including innate and adaptive immune systems, which will give therapeutic benefits for immune-related diseases, such as infection, rheumatoid arthritis, and cancer [16]. *Phyllanthus niruri* water extract increased the proliferation of B cells and T lymphocytes, production of specific cytokines such as TNF-*α*, IFN-*γ*, and IL-4 release. *Phyllanthus niruri* was also able to stimulate macrophage phagocytosis activity and lysosomal enzyme activity and modulate nitric oxide retention by macrophages [17]. Hartini et al. reported that red betel leaf extract and neolignan compounds isolated from these leaves have an immunostimulating effect [18]. Based on the research by Nurrochmad et al., the ethanol extract of *Typhonium flagelliforme* tuber was able to increase the phagocytosis ability of macrophages and the proportion of CD8+ T cells in cyclophosphamide-induced rats [2]. The effect given by these plants is due to their main respective bioactive component. Specifically, pharmacological properties of Piper crocatum Ruiz. and Pav. are widely known that consist eugenol and hydroxychaviol as bioactive components, including eugenol and hydroxychavicol [19], while the main compound in *Phyllanthus niruri* Linn. is phyllanthin [2].

In screening natural products for pharmacological activity, the assessment and evaluation of toxic characteristics of a natural product extract, fraction, or compound are usually an initial step. Regardless of the pharmacological beneficial effects of SKM products, detailed knowledge about the acute and subchronic toxicology of this product is lacking. Hence, the current study evaluated the acute and subchronic toxicity of SKM products in an animal model.

In the acute toxicity study, SKM was administered to test animals in high doses (2000 and 5000 mg/kg b.w.) [20]. There were no significant changes in the central nervous system, somatic motor system, autonomic nervous system, digestive system, skin and hair, mucous membrane, eyes, and organs of the animals during the study.

Mortality is a critical parameter in the toxicity study [21, 22]. In this study, the single administration of SKM product up to 5000 mg/kg b.w. did not cause death or any toxic syndromes in the rats. The acute toxicity test results indicated that the estimated tolerated dose of SKM was greater than 5 g/kg in SD rats. Thus, the LD50 of SKM products was categorized into the unclassified category in accordance with OECD.

The toxicity of SKM was evaluated by performing 90-day subchronic oral toxicity studies in female SD rats. Clinical signs could be used to describe the toxic effects caused by the administration of this product. This study observed no significant changes in general behavior, fur color, eyes, mucous membrane, movement, posture, secretion, and excretion.

Mean body weights were measured to determine the effect of SKM on animal growth. Body weights are closely related to nutrition obtained from food and water consumption. Stress factors could also affect body weight. The decline in body weight gain is an uncomplicated and sensitive indicator after exposure to a substance [23, 24]. Statistical analysis using one-way ANOVA showed no significant differences among the groups (*P* > 0.05), indicating that the administration of SKM did not affect the mean body weights of all animals.

Changes in organ weight can be used as sensitive indices to evaluate substance toxicity [25]. A significant difference in relative kidney weights occurred in the control group. The differences in the relative organ weights were considered an incidental change and did not correlate with the administration of the product because of the lack of differences in the microscopic observation.

No obvious influences on food and water consumption were noted. Changes in food and water consumption occurred in all groups. Food intake could be affected by the individual needs for energy and environmental factors, such as temperature or light cycle [26]. If the environmental temperature is lower than the animal's average temperature, food consumption will increase to maintain a constant body temperature. The factors leading to a decrease in food consumption are high temperature, stress, housing condition, and humidity. The external factor affecting water consumption is the housing condition. High temperature and humidity will decrease water consumption.

Hematological analyses are the essential indices to evaluate the toxicity of substances in humans and animals [27–30]. Evaluation of hematological parameters shows an effect in the blood due to the administration of a test material. No significant differences were found between the dose and control groups, indicating that the product did not affect hematological parameters.

Biochemical parameters are also the fundamental diagnostic criteria in clinical practice. It may indicate the adverse effect caused by substances [31]. Biochemical analyses are used to define, detect, and characterize the toxic effects caused by toxic compounds. The analyses are also crucial for the evaluation of the specific target organ of toxic compounds and provide valuable information for understanding the disease [32]. UREA and CREA values are the parameters used to evaluate kidney damage. The liver function can be evaluated by the levels of markers, including ALT, AST, and TP [29]. The liver and kidney, which are involved in the elimination of xenobiotics, are sensitive organs that can be altered by substances, including plants and drugs [32]. The significant difference between the control group and TP is considered as an individual variation because no changes were found in the histopathological observation. The levels of UREA, CRE, AST, ALT, TP, TC, and GLU were not affected by SKM compared with the control group. The urine examination also did not show any change in the group. Those data indicated that SKM did not alter the functions of the kidney and the liver. The results of biochemical analyses were confirmed by the histopathological study.

The histopathological study is intended to observe any abnormalities in the gross pathology and organ histopathology [33, 34]. The histopathological evaluation of the vital organs showed that the structures were normal and that no microscopic changes occurred in the lung, heart, kidney, liver, stomach, intestine, or lymph.

## 5. Conclusion

This study analyzed the toxicological profile of SKM. The results suggest that the single administration of SKM is nontoxic to the SD rats. Thus, it is categorized into the unclassified category in accordance with OECD.

The daily administration of SKM for 90 days did not affect the clinical signs, mean body weights, food and water consumption, hematological parameters (WBC count, RBC count, platelet count, MCH, MCHC, MCV, Hb, and HCT), clinical biochemistry (AST, ALT, TP, TC, and GLU), urinalysis, relative organ weights, gross necropsy findings, and organ histopathology of the rats.

## Figures and Tables

**Table 1:** Average relative organ weight of male animals exposed to SKM product acutely.

Groups	Number (*n*)	Average of relative organ weight (g)					
		Heart	Lung	Stomach	Liver	Kidney	Lymph
Control	6	0.38 ± 0.03	0.76 ± 0.06	0.77 ± 0.09	3.22 ± 0.34	0.64 ± 0.04	0.28 ± 0.05
SKM 2000 mg/kg b.w.	6	0.32 ± 0.02	0.85 ± 0.13	0.86 ± 0.04	3.72 ± 0.15	0.73 ± 0.06	0.31 ± 0.03
SKM 5000 mg/kg b.w.	6	0.32 ± 0.02	0.71 ± 0.07	0.87 ± 0.10	3.30 ± 0.24	0.62 ± 0.02	0.27 ± 0.02

**Table 2:** Average relative organ weight of female animals exposed to SKM product acutely.

Groups	Number (*n*)	Average of relative organ weight (g)					
		Heart	Lung	Stomach	Liver	Kidney	Lymph
Control	6	0.36 ± 0.01	0.9 ± 0.09	0.85 ± 0.09	3.41 ± 0.19	0.66 ± 0.04	0.29 ± 0.02
SKM 2000 mg/kg b.w.	6	0.37 ± 0.01	0.98 ± 0.11	0.91 ± 0.03	3.44 ± 0.14	0.75 ± 0.02	0.31 ± 0.04
SKM 5000 mg/kg b.w.	6	0.36 ± 0.01	0.82 ± 0.09	0.92 ± 0.12	4.04 ± 0.18	0.65 ± 0.02	0.33 ± 0.01

**Table 3:** Hematological parameters of female SD rats fed exposed to SKM product (22.5; 45; 90 mg/kg b.w.) by oral route and control rats during the 90-day followed by a 28-day recovery period. Values are mean ± SE.

Parameters	Unit	Day 90 (±SEM)				Day 118 (±SEM)		Reference range [13, 14]
		Groups						
		Control	22.5 mg/kg BW	45 mg/kg BW	90 mg/kg BW	Control	90 mg/kg BW	
		*n* = 7	*n* = 9	*n* = 7	*n* = 7	*n* = 5	*n* = 3	
RBC	10^4^/*μ*L	630.71 ± 30.74	702.44 ± 26.98	650.57 ± 20.41	590.00 ± 40.65	696.80 ± 5.39	678.67 ± 33.14	570.00–900.00
WBC	10^2^/*μ*L	59.14 ± 10.96	80.89 ± 11.31^b^	38.43 ± 3.46	52.43 ± 5.55	70.4 ± 8.29	63 ± 11.60	20.00–115.00
Hb	g/dL	12.67 ± 0.51	13.90 ± 0.57	12.99 ± 0.36	11.44 ± 0.79	13.90 ± 0.26	12.80 ± 0.61	11.00–17.00
HCT	%	36.37 ± 1.77	40.44 ± 1.46^a^	38.32 ± 1.11	33.2 ± 2.31	40.38 ± 0.64	38.37 ± 1.94	39.00–55.00
MCV	fL	57.69 ± 0.35	57.63 ± 0.32	58.94 ± 0.46	56.24 ± 0.79	57.96 ± 0.58	56.56 ± 0.54	55.00–65.00
MCH	Pg	20.16 ± 0.27	19.78 ± 0.28	19.99 ± 0.21	19.40 ± 0.34	19.96 ± 0.26	18.87 ± 0.07	17.00–22.00
MCHC	g/dL	34.91 ± 0.38	34.33 ± 0.42	33.89 ± 0.22	34.49 ± 0.16	34.44 ± 0.34	33.37 ± 0.47	28.00–34.00
Platelet	10^4^/*μ*L	27.56 ± 5.13	51.26 ± 7.72	20.51 ± 2.44	36.03 ± 6.71	36.54 ± 7.94	27.90 ± 7.44	70.00–150.00

^a^Significant differences compared with 90 mg/kg b.w. (day 90); ^b^significant differences compared with 45 mg/kg b.w. (day 90).

**Table 4:** Biochemical parameters of female SD rats fed exposed to SKM product (22.5; 45; 90 mg/kg b.w.) by oral route and control rats during the 90-day followed by a 28-day recovery period. Values are mean ± SE.

Parameters	Unit	Day 90 (±SEM)				Day 118 (±SEM)		Reference range [13, 14] (Nurrochmad et al. [2])
		Groups						
		Control	22.5 mg/kg BW	45 mg/kg BW	90 mg/kg BW	Control	90 mg/kg BW	
		*n* = 7	*n* = 9	*n* = 7	*n* = 7	*n* = 5	*n* = 3	
TP	g/dL	9.18 ± 0.18	8.66 ± 0.22	8.57 ± 0.16	8.33 ± 0.21^#^	7.92 ± 0.34	8.13 ± 0.58	5.5–7.3
GLU	mg/dL	93.61 ± 5.69	93.67 ± 7.00	81.54 ± 8.00	81.36 ± 8.00	64.52 ± 7.01	68.43 ± 2.39	70–125 mg/dL
TC	mg/dL	83.31 ± 6.01	74.37 ± 3.64	85.11 ± 3.62	72.49 ± 3.62	64.80 ± 3.31	72.87 ± 2.77	42–90 mg/dL
AST	U/L	138.19 ± 7.51	135.13 ± 8.51	128.44 ± 8.72	149.30 ± 16.00	161.12 ± 35.90	70.37 ± 16.33	80–250 U/L
ALT	U/L	60.80 ± 6.83	63.96 ± 6.26	55.11 ± 3.66	48.90 ± 2.03	71.4 ± 9.58	38.00 ± 8.15	25–50 U/L
UREA	mg/dL	53.27 ± 3.65	49.11 ± 2.45	58.21 ± 3.71	61.04 ± 1.29	31.68 ± 3.08	40.13 ± 1.42	15.1–41.5
CRE	mg/dL	0.48 ± 0.02	0.55 ± 0.04	0.48 ± 0.04	0.41 ± 0.02	0.45 ± 0.03	0.47 ± 0.04	0.3–0.5

**Table 5:** Urinalysis parameters of female SD rats fed exposed to SKM product (22.5; 45; 90 mg/kg b.w.) by oral route and control rats during the 90-day followed by a 28-day recovery period. Values are mean ± SE.

Parameters	Unit	Day 90 (±SEM)				Day 118 (±SEM)	
		Groups					
		Control	22.5 mg/kg b.w.	45 mg/kg b.w.	90 mg/kg b.w.	Control	90 mg/kg
		*n* = 7	*n* = 9	*n* = 7	*n* = 7	*n* = 5	*n* = 3
Urine volume	mL	9.83 ± 5.14	12.44 ± 9.20	14.43 ± 9.75	8.49 ± 4.21	3.46 ± 2.60	1.53 ± 0.92
pH		8.50 ± 0.30	8.40 ± 0.50	9.50 ± 0.50	9.40 ± 0.60	8.80 ± 0.30	8.70 ± 0.30

**Table 6:** Relative organ weight parameters of female SD rats fed exposed to SKM product (22.5; 45; 90 mg/kg b.w.) by oral route and control rats during the 90-day followed by a 28-day recovery period. Values are mean ± SE.

Organ	Relative organ weight (g) ± SE				Satellite relative organ weight (g) ± SE	
	Control (*n* = 7)	Group dose 22.5 mg/kg b.w. (*n* = 9)	Group dose 45 mg/kg b.w. (*n* = 7)	Group dose 90 mg/kg b.w. (*n* = 7)	Control (*n* = 5)	Group dose 90 mg/kg b.w. (*n* = 3)
Lung	0.010 ± 0.002	0.008 ± 0.001	0.006 ± 0.000^∗^	0.010 ± 0.002	0.007 ± 0.000	0.007 ± 0.001
Spleen	0.002 ± 0.000	0.002 ± 0.000	0.003 ± 0.000	0.003 ± 0.000	0.003 ± 0.001	0.004 ± 0.000
Stomach	0.006 ± 0.001	0.006 ± 0.000	0.006 ± 0.000	0.007 ± 0.000	0.007 ± 0.001	0.007 ± 0.001
Heart	0.003 ± 0.000	0.003 ± 0.000	0.003 ± 0.000	0.003 ± 0.000	0.003 ± 0.000	0.003 ± 0.000
Liver	0.020 ± 0.000	0.030 ± 0.000	0.030 ± 0.000	0.030 ± 0.000	0.030 ± 0.000	0.020 ± 0.000
Kidney	0.006 ± 0.000	0.006 ± 0.000	0.006 ± 0.000	0.006 ± 0.000	0.006 ± 0.000	0.006 ± 0.000

^∗^Significant differences compared to the control group.

## Data Availability

All data generated or analyzed during this study are included within the article.

## Abbreviations

SKM: Combination product of *Piper crocatum* Ruiz. and Pav., *Phyllanthus niruri* Linn., and *Typhonium flagelliforme* (Lodd.) BL ethanolic extract
LD50: Lethal dose 50
Ppm: Parts per million
g: Gram
g/kg b.w.: Gram per kilogram body weight
SD: Sprague–Dawley
*μ*g/mL: Microgram per milliliter
OECD: Organization for Economic Cooperation and Development
RBC: Red blood cells
WBC: White blood cells
Hb: Hemoglobin
HCT: Hematocrit
MCH: Mean corpuscular hemoglobin
MCHC: Mean corpuscular hemoglobin concentration
MCV: Mean corpuscular volume
ALT: Alanine transaminase
ANOVA: Analysis of variance
AST: Aspartate transaminase
TP: Total protein
TC: Total cholesterol
GLU: Blood glucose
CRE: Creatinine
PLT: Blood platelet count.

## Additional Points

*Highlights.* (i) The SKM product exerts immunomodulatory activity. (ii) The SKM product was safe up to 5000 mg/kg b.w. in male and female SD rats. (iii) Long-term oral administration of the SKM product for 90 days does not cause subchronic toxicity.
